# Predictive Value of Comorbid Conditions for COVID-19 Mortality

**DOI:** 10.3390/jcm10122652

**Published:** 2021-06-16

**Authors:** Iosif Marincu, Felix Bratosin, Iulia Vidican, Andra-Cristina Bostanaru, Stefan Frent, Bianca Cerbu, Mirela Turaiche, Livius Tirnea, Madalina Timircan

**Affiliations:** 1 Department of Infectious Diseases, “Victor Babes” University of Medicine and Pharmacy, 300041 Timisoara, Romania; imarincu@umft.ro (I.M.); iulia.georgianabogdan@gmail.com (I.V.); frentz.strefan@umft.ro (S.F.); ionitabiancaelena@yahoo.com (B.C.); mirela.turaiche@gmail.com (M.T.); liviustirnea@yahoo.com (L.T.); 2 Laboratory of Antimicrobial Chemotherapy, “Ion Ionescu de la Brad” University of Agricultural Sciences and Veterinary Medicine of Iasi, 700490 Iasi, Romania; acbostanaru@gmail.com; 3 Department of Gynecology, “Victor Babes” University of Medicine and Pharmacy, 300041 Timisoara, Romania; timircan.madalina@yahoo.com

**Keywords:** mortality risk, COVID-19, prediction model, SARS-CoV-2

## Abstract

In this paper, we aim at understanding the broad spectrum of factors influencing the survival of infected patients and the correlations between these factors to create a predictive probabilistic score for surviving the COVID-19 disease. Initially, 510 hospital admissions were counted in the study, out of which 310 patients did not survive. A prediction model was developed based on this data by using a Bayesian approach. Following the data collection process for the development study, the second cohort of patients totaling 541 was built to validate the risk matrix previously created. The final model has an area under the curve of 0.773 and predicts the mortality risk of SARS-CoV-2 infection based on nine disease groups while considering the gender and age of the patient as distinct risk groups. To ease medical workers’ assessment of patients, we created a visual risk matrix based on a probabilistic model, ranging from a score of 1 (very low mortality risk) to 5 (very high mortality risk). Each score comprises a correlation between existing comorbid conditions, the number of comorbid conditions, gender, and age group category. This clinical model can be generalized in a hospital context and can be used to identify patients at high risk for whom immediate intervention might be required.

## 1. Introduction

Previously known as 2019-nCOV, the novel coronavirus (SARS-CoV-2) is the causal agent of the coronavirus disease 2019, or COVID-19 pneumonia, as named by the WHO [1]. First reports of the disease date to December 2019, from an outbreak of pneumonia of unknown cause, originating in Wuhan, China [2]. The certified source of contamination is yet to be identified to this day. The virus is responsible for infections in all age categories, having an airborne transmission type [3]. Clinical characteristics of COVID-19 are non-specific, including the majority of signs and symptoms related to respiratory infections, most commonly fever, cough, and fatigue [4], and more specifically, including the loss of smell and taste senses [5]. At the same time, nasal congestion and rhinorrhea are rare [6].

Case fatality rates for the SARS-CoV-2 confirmed patients range from higher values such as 8.8% in Mexico to as low as 0.3% in the United Arab Emirates [7], with an average of 2–3% worldwide [8]. This rate can be influenced by country demographics, region-specific characteristics, infection curve shapes, the healthcare system, and preventive measures taken by each country. Currently, the COVID-19 patients in Romania are being admitted to the hospital based on the severity of the illness and their access to care at home. Various treatment schemes are implemented based on three illness categories (mild, medium, and severe), including antivirals such as remdesivir, dexamethasone as corticosteroid, empiric antibiotic treatment, anticoagulants like nadroparin, and other associations based on patients’ comorbidities.

This research aims to create and validate a simplified mortality prediction model based on a spectrum of the most frequently encountered diseases, comprised of 13 wide categories of the most commonly found pathologies in the general population, using the TRIPOD statement as a referencing guideline [9]. The model aims for better-distributed attention to care for high-risk cases while also facilitating the triage of confirmed SARS-CoV-2 infections into early hospital admissions or stay-at-home for those who are found to be at low mortality risk, based on the assessment of existing comorbidities. The study describes the development of the prognostic score and its internal validation in a real-life scenario.

## 2. Methods

### 2.1. Building the Prediction Model

Data was collected from the “Victor Babes” Infectious Diseases and Pulmonology Hospital from Timisoara from April 2020 to September 2020. The initial data collection was conducted to build the risk prediction model, which would later be used in a prospective cohort for validation. The initial study arrived at a sample of 510 patients, from which 200 were discharged as asymptomatic from our department after three consecutive negative RT-PCR tests, while the remaining 310 patients did not survive the infection. The eligibility criteria for participants included confirming SARS-CoV-2 infection using the RT-PCR method, as well as the eligible patients accepting the enrollment into our study. No other specific criteria were taken into account. The SARS-CoV-2 treatment has not been modified during the study period, as all participants received a treatment scheme recommended at the time of the study by the Romanian Ministry of Health, including the antiretroviral therapy with lopinavir/ritonavir, hydroxychloroquine, an empiric broad-spectrum antibiotic with ceftriaxone or moxifloxacin, and anticoagulation with nadroparin. No corticoids have been used in these patients. To check the performance and validity of our prediction model, we opted for an internal validation method using an Operating Characteristic Curve [10]. Thus, the prediction model was tested on a new cohort of patients in the following 3 months after the initial data collection. The aim was to run the patients’ assessment using the proposed risk matrix and compare the outcomes with the prediction. After receiving the confirmation of SARS-Cov-2 infection, each patient was given a choice to be assessed using the prediction model and informed consent to be signed as proof of willingness to participate in the study.

### 2.2. Outcome

The proposed prediction model should be able to give a reliable estimate of COVID-19 mortality risk using a ranking score based on the computed probability of surviving the illness. It has to be assessed after the PCR test confirms the SARS-CoV-2 infection, while the physician is collecting background information regarding the patient’s comorbid conditions, age, and gender. After the test is confirmed and relevant medical history is collected, the physician will then look over the risk matrix to check for the probability of survival for the patient being assessed. The model started initially with 15 predictors, including the patient’s age and gender, and 13 groups of diseases identified as: malignancy, lung disease, hypertension, diabetes mellitus, heart disease, kidney disease, liver disease, obesity, autoimmune disease, recent surgery, neurologic disorders, stroke, and hematology disturbances. No actions for blinding the predictors were performed.

### 2.3. Predictors

The inclusion criteria for malignancy comprises all solid malignant tumors. Lung disease was defined by the following spectrum of conditions: chronic obstructive pulmonary disease (COPD), asthma, pneumothorax, pulmonary hypertension, pneumonia (of other causes than SARS-Cov-2 infection), and tuberculosis. Hypertension includes primary or secondary systemic elevated blood pressure. All patients had either type 1 or type 2 diabetes mellitus. The heart disease group of comorbidities includes heart failure, old or recent myocardial infarction, chronic heart arrhythmias, valvular disease, cardiomyopathies, and ischemic heart disease. In this study, patients with kidney disease had either chronic kidney disease (CKD) or acute renal failure. Liver disease is comprised of cirrhosis and hepatitis (infectious or toxic). Obesity is defined by a body mass index (BMI) equal to or higher than 30 kg/m^2^. Autoimmune diseases found in our sample include thyroiditis, rheumatoid arthritis, systemic lupus erythematosus (SLE), and psoriasis. Recent surgery is defined by patients whose SARS-CoV-2 infections occurred during the hospital stay for surgery. The neurologic disorder group of comorbidities comprises the following: neurologic deficit, multiple sclerosis, Parkinson’s disease, Alzheimer’s dementia, and encephalopathy. The stroke group includes patients whose SARS-CoV-2 infections occurred while admitted to the hospital for a stroke. Finally, hematologic disturbances found in this sample include leukemias, lymphomas, multiple myeloma, and anemia of all causes.

### 2.4. Risk Groups

Based on literature [11], gender was considered the first risk group, where women seemed to have a better prognosis. On the same idea, three age groups were proposed as significant risk factors, where age was proved to influence the survival rates for COVID-19 infection [12], with an increase in case fatality ratio from 0.2% in patients younger than 19, up to 14.8% in patients older than 80. Age groups were defined as below 30 years old (yo) patients (<30 yo), between 30 and 60 years old patients (30–60 yo), and older than 60 years patients (>60 yo).

### 2.5. Statistical Analysis Methods

Data were analyzed using IBM SPSS Statistics version 26 for Windows operating system. Mann–Whitney U-test was used for the continuous variables, while the Chi-square test was used for the categorical data. The Kruskal–Wallis H-test was used to test differences between the proposed age groups. The statistically significant predictors were introduced in a linear regression model, while variables found without a statistically significant prediction value were excluded from the model using a backward elimination approach. Finally, the remaining predictors were tested for collinearity, after which a naïve Bayesian model was constructed to assess probabilities. All statistics were tested on a 99% significance threshold. A receiver operating characteristic curve (ROC) was built with the information collected for internal validation to assess our model’s performance while having to predict the probability of a binary outcome such as surviving or not surviving the SARS-Cov-2 infection. Thus, the ROC and area under the curve (AUC) were plotted and calculated.

## 3. Results

### 3.1. Participants

The development sample (Table 1) included a total of 510 patients, out of which 310 did not survive the infection. In the non-survivors group, the mean age was 67 years, and 61.9% were men. The most common condition in this group was high blood pressure, seen in 113 patients, followed by heart disease in 121 patients, and 106 cases of diabetes mellitus. On the other side, the group of survivors comprised 91 (43.3%) men and 109 women, with a mean age of 49 years. None of these 200 patients suffered from malignancies or hematological disturbances. As well as in the non-survivors group, arterial hypertension was the most encountered comorbidity, followed by diabetes in 37 patients, and heart disease in 20 patients. Mentioning these two lists, a patient can have more than one condition at a time. There were no statistically significant differences between survivors and non-survivors comparing hypertension (*p*-value 0.063), autoimmune disease (*p*-value 0.312), and recent surgery (*p*-value 0.13).

### 3.2. Model Development

A statistically significant difference was found between the survival rates in men and women (*p*-value = 0.0002; Cramer’s V = 0.161; OR female/male = 0.513), thus, opting for creating a risk grouping by gender. An analysis was performed for age groups (Kruskal–Wallis test *p*-value < 0.00001), and between-groups Mann–Whitney test (*p*-value = 0.005 between <30 yo and 30–60 yo; *p*-value < 0.00001 between <30 yo and >60 yo; *p*-value = < 0.00001 between 30–60 yo and >60 yo), thus, opting for a secondary risk grouping by breaking down our sample to three age groups.

### 3.3. Model Specification

A score of 1 is attributed to a probability of not surviving the COVID-19 infection of less than 10%. Patients with a probability between 10% and 30% of not surviving the illness are given a score of 2. For a probability between 31% and 50%, the patients are given a score of 3. A score of 4 is given for a 51 to 70% probability of death, while those with very high chances of not surviving the infection (>70%) are given a score of 5 (Table 2).

### 3.4. Model Performance

**Figure 1:**
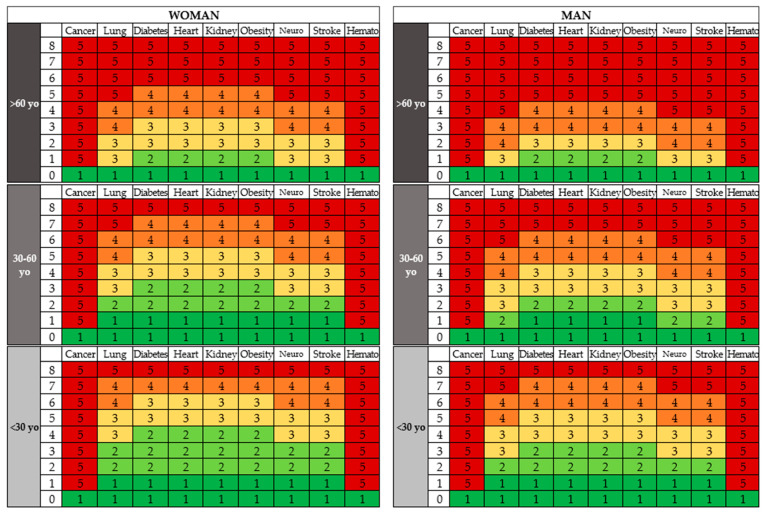
The prediction matrix.

A logistic regression model was built after excluding hypertension, autoimmune disease, and recent surgery from the list of significant risk factors (Table 3). Liver disease was later excluded from the model, considering an insignificant prediction power (OR 0.7–16.9). The physician will take the patient’s medical history and then calculate the prediction score based on the colored two-dimensional risk matrix (Figure 1). Assessing the patient’s risk score based on the prediction matrix is a three-step process where the physician selects one of the six boxes on the matrix that the patient falls into by doing the following: (1) based on the patient’s gender, the physician will look to the left if the patient is a woman, or to the right for a male patient; (2) based on the patient’s age, the physician will look on the vertical axis to the age group where the patient fits (<30 yo, 30–60 yo, or >60 yo); (3) the doctor counts the patient’s comorbidities on the horizontal axis, then checks which group of diseases has the highest risk of which the patient belongs on the vertical axis. By uniting the two axes, the physician will get a score ranging from 1 to 5.

Out of 541 patients involved in the validation study (Table 4), 299 survived, while the other 242 died. A total of 37 patients were predicted in the <10% mortality risk group, out of which 3 (8%) have died. Eighty-five patients were predicted in the 10–30% mortality risk group, while 21 (24%) died. A number of 113 patients were predicted to fall in the 31–50%, and 35 (31%) died. A number of 187 patients were predicted to have between 51% and 70% mortality risk, out of which 97 (52%) died. Lastly, 119 were predicted to have a greater than 70% risk of COVID-19 mortality, while 86 (72%) did not survive.

**Figure 2:**
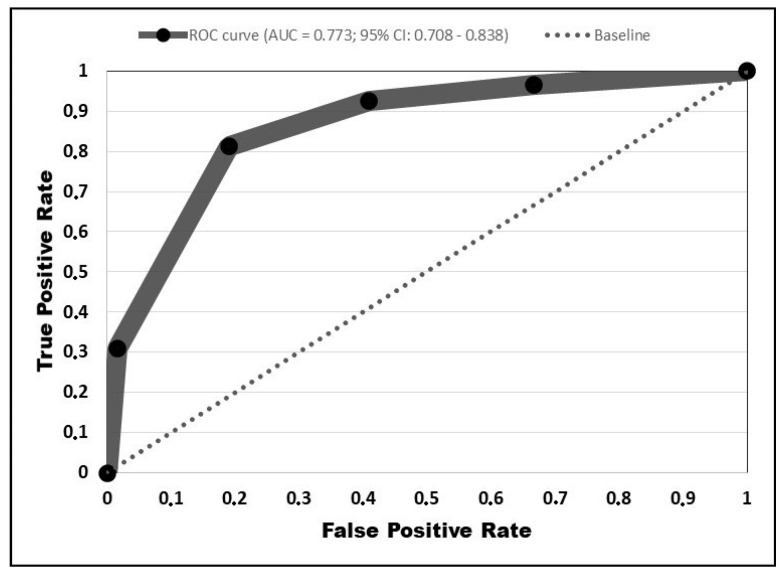
Receiver operating characteristic curve.

The ROC curve was plotted (Figure 2), with the AUC = 0.773 (95% CI: 0.708–0.838). Based on the AUC value, our predictive risk matrix is considered to have an acceptable accuracy [13]. Thus, there is a 77% chance that the physician reading the risk matrix while conducting the patient assessment will correctly predict an outcome.

## 4. Discussion

### 4.1. Interpretation

When reading the matrix, gender will be the first criteria to be taken into consideration, then the age group of the patient. After one of the six main blocks on the matrix has been established, the physician/healthcare worker will check the comorbidity with the highest risk of not surviving the infection and count the number of associated comorbid conditions from a total of 8, thus obtaining the respective risk score.

### 4.2. Implications

Recently published research suggests early initiation of treatment with remdesivir, since it was proven to significantly reduce viral titers if given before the peak viral replication happens [14]. On the contrary, other studies [15] criticize the efficacy of remdesivir due to inconsistent results in trials that were underpowered. Thus, our prediction model helps in identifying patients at risk after testing positive and early use of remdesivir if the prediction suggests [16] before the clinical presentation of symptoms. The model is set in hospitalized cases and can be generalized in a hospital context. The early initiation of treatment for hospitalized patients based on our prediction model will benefit not only the patient but also the hospital since a shorter duration of stay facilitates the management of supplies and the availability of hospital beds. The triage process is improved by quickly calculating a prediction in a setting of hundreds or thousands of daily confirmed cases as it only involves taking a quick patient history and finding the broad category of comorbidities that a respective patient is suffering from.

### 4.3. Limitations

Risk prediction models can be overfitting by assuming to behave conservatively in the data pool on which they are built relative to the performance observed when measured in a new setting, yet with comparable individuals [17]. This is a result of the model being built to maximally match the development sample but becomes less reliable when evaluated in new but identical individuals.

The collected sample is homogenous and shares the characteristics of Romanian demographics, making the risk assessment chart arguably compatible with regions and countries that share different characteristics. For example, a study [18] included the health system capacity as a prediction factor, determining that Eastern Europe as a region has a greater infection fatality rate (IFR). Thus, reports of COVID-19 IFRs in Europe are considered to differ by age, sex, and comorbidity. In the same category, the preparedness of a country’s health system and equipment availability and trained physicians might represent independent risk factors for COVID-19 mortality. Additionally, reports observe a racial disproportion, where African Americans are more likely to die of COVID-19 infection by 3–6 times [19]. In such conditions, the proposed predictive score might be inappropriately used with an African American patient.

The survival rate in the development sample was 39.2%, compared to the 55.2% in the validation cohort. Such differences could be attributed to the pandemic evolution in Romania. The development sample was collected during a wave peak when our clinic admitted more severe cases due to the availability of hospital beds. On the other side, the validation cohort overlapped a period of lower case-incidence. There was no improvement in therapies during the study period that might influence the difference in patient survival rates observed between the two cohorts.

With several exceptions, including diabetes mellitus, hypertension, obesity, and stroke, the other groups of diseases included in the risk matrix comprise a wide spectrum of pathologies based on organ systems rather than a specific diagnosis, thus raising the likelihood of a certain disease comprised in the wider group to be an independent risk factor. Moving to a greater extent in discovering highly specific predictors for COVID-19 mortality, a considerable number of studies focus on evaluating hematologic abnormalities as they are thought to be more accurate indicators for disease severity and mortality risk. These factors were not particularly included in our prediction model, although the “hemato” rubric on our risk matrix considers all abnormalities discovered in each patient evaluated. Here, a systematic review carried by Shahri et al. [20] discovered that leukopenia seems to be directly proportional correlated to COVID-19 disease severity, while lymphopenia can be used as a prognostic prediction factor. Another systematic review conducted by Slomka et al. [21] proves that D-dimer levels are markedly increased in patients with severe COVID-19 infection. In contrast, platelet levels are significantly decreased in those admitted to the intensive care unit.

Although the COVID-19 mortality averages from 3% to 5%, our clinic registered a higher overall mortality rate in the number of patients received due to the admission of severe cases, since our facility is among the best-equipped in the Western Romanian territory. Here, the findings are consistent with other studies involved in researching the risk factors for COVID-19 mortality. A multicentric study developed in Romania at the beginning of the pandemic [22] identified that male gender, hypertension, obesity, chronic kidney disease, and diabetes are responsible for a worse probability of surviving the SARS-Cov-2 infection. On the same level, the CDC [23] has created a list of underlying medical conditions that are currently known to be responsible for significantly increasing the risk of a severe form of COVID-19, based on scientific evidence. Here, as well as our study concluded, the CDC included malignancy, renal disease (CKD), pulmonary disease (COPD), heart conditions, obesity, hematological disturbances (sickle cell disease), in the list of significant risk factors for COVID-19 mortality.

## Figures and Tables

**Table 1:** Key characteristics of the development sample.

Characteristic	Non-Survivors (*n* = 310)	Survivors (*n* = 200)	*p*-Value
Mean age (range) *	67 (27–98)	49 (18–90)	<0.00001
Male	192 (61.9)	91 (45.5)	-
Female	118 (38)	109 (51.9)	-
Malignancy	24 (7.7)	0 (0.0)	0.00005
Lung disease	57 (18.3)	7 (3.5)	<0.00001
Hypertension	113 (36.4)	57 (28.5)	0.063
Diabetes	106 (34.2)	37 (18.5)	0.0001
Heart disease	121 (39)	20 (10.0)	<0.00001
Kidney disease	52 (16.8)	6 (3.0)	<0.00001
Liver disease	40 (12.9)	4 (2.0)	0.00002
Obesity	45 (14.5)	13 (6.5)	0.005
Autoimmune disease	13 (4.2)	5 (2.5)	0.312
Recent surgery	13 (4.2)	1 (0.5)	0.013
Neurologic disorders	53 (17)	5 (2.5)	<0.00001
Stroke	27 (8.7)	3 (1.5)	0.001
Hematological disturbances	19 (6.1)	0 (0.0)	0.0003

* Values are *n* (%) unless indicated differently.

**Table 2:** Specifications of the mortality risk prediction matrix.

Probability	Risk	Score
<10%	Very Low	1
10–30%	Low	2
31–50%	Medium	3
51–70%	High	4
>70%	Very High	5

**Table 3:** Risk matrix predictors.

Comorbid Condition	*p*-Value	OR (99% CI)
Malignancy	0.003	7.6 (1.1–19.6)
Lung disease	0.0003	5.1 (1.5–16.5)
Diabetes mellitus	0.001	2.3 (1.1–4.4)
Heart disease	<0.0001	5.6 (2.6–11.8)
Kidney disease	0.0004	5.5 (1.5–19.4)
Liver disease	0.03	3.6 (0.7–16.9)
Obesity	0.003	3.1 (1.1–8.0)
Neurological disorders	0.0001	7.4 (1.9–27.7)
Stroke	0.002	7.9 (1.4–43.3)
Hematology disturbances	0.0001	8.4 (1.4–3.3)

**Table 4:** Characteristics of the validation study.

Predicted	Lives	Dies	Result
<10%	34	3	8%
10–30%	64	21	24%
31–50%	78	35	31%
51–70%	90	97	52%
>70%	33	86	72%

## Data Availability

The data presented in this study are available on request from the corresponding author. The data are not publicly available due to privacy restrictions.
